# From Bovine Immune Milk Profiling to Multi-Antigen Vaccine Design: Enhanced Humoral Responses Against *H. pylori* with a Flagellin and Urease Subunit Cocktail

**DOI:** 10.3390/vaccines14020110

**Published:** 2026-01-23

**Authors:** Hongru Li, Enhao Zhang, Jingyuan Ning, Yushan Lin, Guanyuan Wang, Hong Zhang, Cuixia Ma, Jiachao Wang, Miao Li, Xue Gao, Chenhui Li, Lin Wei, Xian Wang, Cuiqing Ma

**Affiliations:** 1 Key Laboratory of Immune Mechanism and Intervention on Serious Disease in Hebei Province, Department of Immunology, Hebei Medical University, Shijiazhuang 050017, China; lihongru@stu.hebmu.edu.cn (H.L.); 24033100062@stu.hebmu.edu.cn (E.Z.); ninglab@163.com (J.N.); 23033100057@stu.hebmu.edu.cn (Y.L.); 24033100035@stu.hebmu.edu.cn (G.W.); macuixia0512@sina.com (C.M.); 19001550@hebmu.edu.cn (J.W.); 18200739@hebmu.edu.cn (M.L.); 18600827@hebmu.edu.cn (X.G.); weilin@hebmu.edu.cn (L.W.); 2 Hebei Key Laboratory of Intractable Pathogens, Shijiazhuang Center for Disease Control and Prevention, Shijiazhuang 050011, China; zhsjzcdc@126.com; 3 Department of Genetics, Shijiazhuang Maternity and Child Healthcare Hospital, Shijiazhuang 050051, China; 4 Hebei Key Laboratory of Autoimmune Disease Medicine Research, Shijiazhuang 050035, China; li.chen.hui@163.com

**Keywords:** *Helicobacter pylori*, Immune milk, multi-antigen immunogen, humoral immunity, non-antibiotic therapy

## Abstract

Objective: The aim of this study was to develop and evaluate non-antibiotic strategies against *Helicobacter pylori* by establishing a bovine immune milk platform and designing a synergistic multi-antigen immunogen to enhance humoral immune responses. Methods: Inactivated *Helicobacter pylori* (*H. pylori)* was used to immunize dairy cows, and the resulting immune milk was characterized for antibody specificity, acid stability, and target antigens via ELISA, Western blot, agglutination assays, and mass spectrometry. Key identified antigens (UreA, UreB, UreE, UreG, HypA, FlaA, and FlaB) were produced as recombinant proteins. Their immunogenicity was evaluated in a murine model, comparing single antigens with various protein combinations. Immune responses were assessed by antigen-specific IgG ELISA, bacterial agglutination titers, flow cytometry for T-cell activation, and histopathology for safety. Results: Immune milk contained high-titer, acid-stable IgG antibodies targeting multiple *H. pylori* virulence factors. In mice, while single proteins induced specific IgG, a multi-antigen cocktail (FlaA + FlaB + HypA + UreA + UreB + UreE + UreG) elicited significantly higher serum agglutination titers (~7 × 10^3^) than single antigens or inactivated whole-cell vaccine, alongside robust CD4^+^ T-cell activation. No formulations showed any hepatorenal or splenic toxicity. Conclusion: Bovine immune milk is a viable platform for acid-stable antibody delivery. A rationally designed multi-antigen cocktail synergistically enhances functional humoral immunity in vivo, providing a promising foundation for developing antibody-based or subunit vaccine strategies against *H. pylori*.

## 1. Introduction

*H. pylori*, a Gram-negative, microaerophilic bacterium colonizing the human gastric mucosa, represents a major global health burden [1] due to its role in chronic gastritis, peptic ulcer disease, and gastric cancer [2,3,4]. Despite the availability of antibiotic-based eradication regimens, increasing antimicrobial resistance and the adverse impact on gut microbiota underscore the urgent need for effective non-antibiotic alternatives [5,6,7].

The pathogenicity of *H. pylori* is intimately linked to its repertoire of virulence factors, which facilitate gastric colonization, immune evasion, and tissue damage. Among these, urease, constituting 6–15% of bacterial protein, is essential for acid acclimatization and colonization [8]. Flagellar proteins FlaA and FlaB are critical for motility and penetration of the gastric mucus layer. Additionally, effectors like CagA and VacA contribute to host cell modulation and immune evasion [9].

Passive immunotherapy with pathogen-specific antibodies presents a promising therapeutic avenue. Historically, immune bovine milk has demonstrated efficacy against various enteric pathogens, including rotavirus, Escherichia coli, Streptococcus pyogenes, and Cryptosporidium spp. [10,11]. Notably, early studies indicated that anti-*H. pylori* antibodies in milk could exert bactericidal activity in vitro [12], and maternal milk containing anti-urease antibodies delayed *H. pylori* colonization in infants [13]. Despite these advances, clinical trials using bovine anti-*H. pylori* milk induced by inactivated vaccine achieved only moderate clearance rates (33.3%) [14], highlighting the need for optimized immunogens to elicit broader and more potent antibody responses.

To address this, we conducted a two-phase investigation in this study. First, we produced and characterized *H. pylori*-specific immune milk from cows immunized with inactivated whole bacteria, confirming the stability and gastric acid resilience of the resulting IgG, and identifying key virulence factors by these antibodies targeted, including urease subunits (UreA, UreB, UreE, UreG, and HypA) and flagellins (FlaA and FlaB) by proteomic analysis. Second, we expressed and systematically evaluated recombinant urease-associated proteins and flagellins as subunit vaccine candidates in a murine immunization model. We demonstrate that a multi-protein cocktail combining flagellins with urease subunits induces synergistic humoral immune responses, surpassing the agglutination titers elicited by single antigens or traditional inactivated whole-cell vaccines.

## 2. Materials and Methods

### 2.1. Bacteria and Experimental Animals

The *H. pylori* strain utilized in this experiment was NCTC11637, maintained at the Center for Disease Control and Prevention in Shijiazhuang. Female BALB/c mice, aged 6 to 8 weeks, were housed within the animal facilities of Hebei Medical University. All murine experiments were conducted in strict compliance with the Guide for the Care and Use of Laboratory Animals, adhering to the 3Rs principle (Replacement, Reduction, Refinement), and the animal protocol was approved by the Ethics Committee for Animal Experiments at Hebei Medical University (License No. IACUC-Hebmu-P 2022065) to ensure the welfare of experimental mice.

### 2.2. Construction, Expression, and Purification of Recombinant Proteins

The construction, expression, and purification of recombinant proteins were performed as previously described with some modifications [15]. Briefly, genes encoding UreA (GenBank: ADM51993.1), UreB (GenBank: AKE47408.1), UreE (GenBank: AAA25023.1), UreG (GenBank: AAA25025.1), HypA (GenBank: ACX97671.1), FlaA (GenBank: AGI42755.1), and FlaB (GenBank: AGI42752.1) were amplified by PCR using the genes of *H. pylori* strain as templates, and inserted into the pET28a(+) expression vector (Yeasen, Shanghai, China). Successful construction of the recombinant plasmids was confirmed by sequencing. The recombinant plasmids were then transformed into *E. coli* BL21 (DE3) competent cells (Biyun Tian, Shanghai, China. No.D1013S). Protein expression was induced using isopropyl-β-D-thiogalactopyranoside (IPTG) at a final concentration of 0.5 mM; subsequently, the recombinant proteins were purified utilizing a His-tagged Protein Purification Kit (P2226, BiyunTian) according to the manufacturer’s instructions.

The purified proteins were analyzed by SDS-PAGE, and protein concentrations were determined using the BCA method.

### 2.3. ELISA

The titers of specific IgG and IgA antibodies against *H. pylori* in the milk from immunized cows were detected using the enzyme-linked immunosorbent assay (ELISA) as previously described with some modifications [16]. In brief, serial dilutions of either immunized or normal milk were added to a 96-well microtiter plate that had been pre-coated with heat-inactivated *H. pylori* (10 μg/mL) and blocked with 2% non-fat milk. The plates were incubated at 37 °C for 1 h and subsequently washed four times with phosphate-buffered saline containing 0.1% Tween 20 (PBST). Then the conjugated antibody was reacted with horseradish peroxidase (HRP)-coupled anti-bovine IgG (1:3000; Sigma, Saint Louis, Missouri, USA) or anti-bovine IgA (1:2000; Sigma) for one hour at 37 °C. The reaction was visualized by substrate 3,3,5,5-tetramethylbenzidine (TMB) (Invitrogen, Carlsbad, CA, USA) and stopped by 1 N H_2_SO_4_. The absorbance at 450 nm (A450) was measured using an ELISA plate reader (Tecan, Männedorf, Switzerland).

Additionally, specific antibodies against UreA, UreB, UreE, UreG, HypA, FlaA, and FlaB were detected in both immunized and normal milk samples according to the protocol provided by an ELISA kit from Shanghai Zhenke Biotechnology Co., Ltd. (Shanghai, China), as described above.

### 2.4. Immunization of Dairy Cows

The immunization of cows was performed as previously described with some modifications [14]. Briefly, three pregnant dairy cows were immunized with a mixed suspension of *H. pylori* strains Hp5162 and Hp4236, with 8 immunization rounds administered: 2 rounds in the final month prepartum and 6 rounds postpartum. Each immunization dose contained 6 × 10^9^ colony-forming units (cfu) of *H. pylori*, delivered via subcutaneous injection. The immunization interval was standardized at approximately 2 weeks. For the primary immunization, the *H. pylori* suspension was emulsified with an equal volume of Freund’s complete adjuvant; all subsequent booster immunizations utilized Freund’s incomplete adjuvant at the same volume ratio for emulsification.

### 2.5. Detection of Antibody in the Immunized Milk Binding to H. pylori

Magnetic beads coated with protein A/G (Pierce) were co-incubated with immunized milk and *H. pylori* to form complexes of magnetic bead-IgG-*H. pylori*. The magnetic beads were subsequently washed to eliminate non-specifically bound bacteria, after which a gradient of the magnetic beads was applied onto Columbia agar plates. The number of antibody-bound *H. pylori* was then quantified through plate counting methods.

### 2.6. Protein Interaction Network

The Protein–Protein Interaction (PPI) Network comprises individual proteins that engage in interactions with one another. Here, we ultrasonically fragmented strain NTCT11637 and co-incubated Pierce protein A/G magnetic beads with immunized milk and bacterial proteins. Subsequently, we performed immunoprecipitation and conducted protein profiling on the coprecipitated bacterial proteins. We employ the degree algorithm to assess the protein that has the highest correlation with IgG. The PPI network model was visualized using Cytoscape 3.9.1 [17].

### 2.7. Mouse Vaccination and Sample Collection

Mice were initially vaccinated subcutaneously with UreA, UreB, UreE, UreG, HypA, FlaA, or FlaB (10 μg/mouse), following a previously described protocol [18]. Briefly, mice were prime-vaccinated (subcutaneously, s.c.) with 10 μg/mouse of recombinant proteins in the presence of Complete Freund’s Adjuvant (CFA) (Alhydrogel^®^ adjuvant 2%, Invivogen, San Diego, CA, USA) at a volume of 100 μL/mouse. They were then boosted twice with the same immunogen and incomplete Freund’s Adjuvant (IFA) at 2-week (14 days) intervals. Sera from 0, 10, 22, and 34 days post-initial vaccination of each group were detected for recombinant protein-specific antibody response and bacterial agglutination assays. A total of 40 mice were assigned to 8 groups with 5 mice in each group.

Immunization with bacterial protein combinations was based on the ability of these proteins to interact with one another as well as their binding affinities. These combinations included UreA + UreB, UreE + UreG, HypA + UreE, HypA + UreE + UreG, HypA + UreA + UreB + UreE + UreG, and FlaA + FlaB + HypA + UreA + UreB + UreE + UreG (10 μg/protein/mouse), still administered alongside Freund’s Adjuvant (2%, Invivogen). Specific experiments were conducted as described above. A total of 40 mice were assigned to 8 groups (6 groups of different target protein combinations, inactivated vaccine as positive control, and PBS as negative control), with 5 mice assigned randomly in each group. After grouping, the body weight and general health status of mice in each group were statistically analyzed, and no significant differences were found (*p* > 0.05), confirming the randomness and balance of the grouping.

### 2.8. Flow Cytometry

T-cell responses in immunized mice were assessed by flow cytometry as previously described [19]. In brief, splenocytes (2 × 10^6^) were fixed utilizing the Cytofix/Cytoperm™ Plus kit according to the manufacturer’s protocol (BD Biosciences, Franklin Lakes, NJ, USA) and subsequently stained with conjugated anti-mouse CD3 (APC-Cy7), anti-mouse CD4 (FITC), and anti-mouse CD8 (PE-Cy5) for 30 min at 4 °C. Appropriate isotype-matched controls were included in each staining. The stained cells were analyzed using flow cytometry (FACSCalibur; BD Biosciences), and the data were evaluated by FlowJo^®^ v10.0 software (BD Biosciences).

### 2.9. Bacterial Agglutination Assay

The bacterial agglutination assay was employed to detect the neutralizing antibody response in immunized mouse serum. Briefly, sera from immunized mice were collected and mixed with varying concentrations of NCTC11637 *H. pylori* strain suspensions, followed by incubation at room temperature for 30 min. To control background reactions, serum from non-immunized mice served as a negative control, while a positive control group was established. The level of agglutination was assessed under microscope. The agglutination titer was expressed as the reciprocal of the highest dilution of serum at which clear agglutination occurs.

### 2.10. Statistical Analysis

Statistical analysis was performed using Graphpad Prism (version 9.0) software. All data are presented as mean ± standard deviation (SD). Comparisons between two groups (control vs. model group) were assessed using an unpaired, two-tailed Student’s *t*-test. A *p*-value < 0.05 was considered statistically significant.

## 3. Results

### 3.1. Milk from Cows Immunized with Inactivated H. pylori Contains Specific, Acid-Stable IgG Against H. pylori

**Figure 1:**
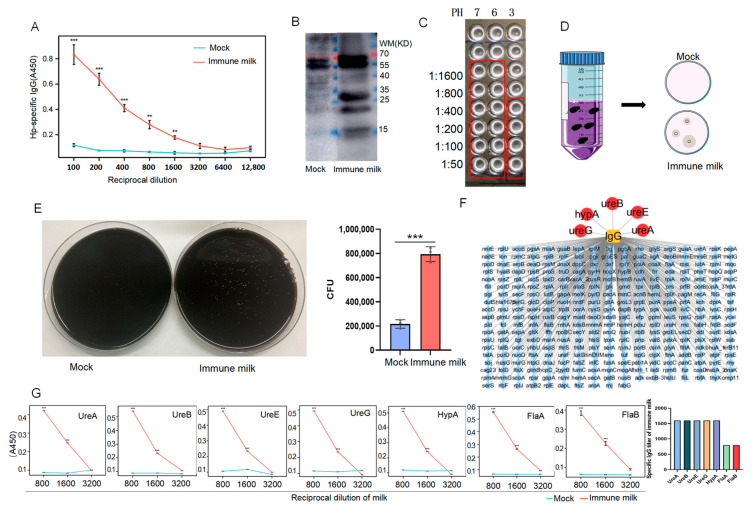
**Milk from immunized cows with inactivated *H. pylori* contains specific IgG against *H. pylori*.** (**A**) Detection of anti-*H. pylori* IgG antibodies in milk by ELISA. (**B**) Western blot detection of antibodies against *H. pylori* in immunized or normal milk. (**C**) Agglutination assays of immune milk and *H. pylori* at PH of 3, 6, and 7. (**D**) Schematic diagram of immunoprecipitation. (**E**) Bar graph of number of *H. pylori* bound to immunized milk through CoIP (*n* = 3). (**F**) Mass spectrometric detection of *H. pylori* cell proteins bound by specific IgG antibodies in immune milk. (**G**) ELISA analysis of immunized milk containing specific IgG against each bacterial protein of *H. pylori* (*n* = 5) (vs. Mock, ** *p* < 0.01, and *** *p* < 0.005).

Unlike human milk, cows’ milk is particularly rich in IgG (both IgG1 and IgG2), with lower levels of IgA and IgM, and the concentration of IgG in mature milk is 200–500 μg/mL, while it reaches 50–100 mg/mL in colostrum [20]. Milk from immunized cows contained high-titer IgG antibodies against *H. pylori* (titer 1:1600), which remained detectable after three years of storage, demonstrating remarkable stability (Figure 1A). However, no detectable levels of IgA were observed (Figure S1A). Furthermore, after ultrasonic disruption of *H. pylori*, we extracted the proteins, performed SDS-PAGE, and transferred the membrane, which was blotted with the immunized milk or normal milk. The results showed that, compared with the mock group, the immunized milk could bind the bacterial proteins particularly significantly (Figure 1B). Subsequently, agglutination assays were performed to evaluate the binding capacity of the antibody-enriched milk to *H. pylori* under both near-neutral and acidic (pH 3) conditions. The results demonstrated a significant agglutination titer of 1:1600 under near-neutral pH, indicating strong antibody–pathogen interaction. Notably, even under the highly acidic condition mimicking the gastric environment, a measurable agglutination titer of 1:400 was retained (Figure 1C), indicating that the IgG antibodies present in the immune milk maintain their functional activity and binding capability to *H. pylori* in a gastric-like environment. Additionally, after incubating the bacteria with the immunized or normal milk, we precipitated the bacteria that were bound to the antibodies in the milk using magnetic beads coated with Pierce protein A/G. The results showed that compared with the control group, the amount of *H. pylori* bound by the immunized milk significantly increased, suggesting that the immunized milk contained a large amount of antibodies targeting the surface components of *H. pylori* (Figure 1D,E).

To further identify the bacterial proteins targeted by the anti-*H. pylori* antibodies in the immunized milk, we ultrasonically fragmented *H. pylori* by performing co-immunoprecipitation with immunized or control milk, and then we detected the protein profiling on the coprecipitated bacterial proteins, which revealed that the antibodies in the immunized milk could bind to a range of bacterial proteins (Figure 1F). Notably, these antibodies specifically bound several urease-related proteins, including UreA, UreB, UreE, and UreG—subunits critical to urease function—as well as the nickel-dependent HypA protein, which is crucial for promoting maturation and activation of urease, and flagellin proteins FlaA and FlaB. Importantly, the titers of IgG in the immune milk against these components, except for FlaA and FlaB, all reached 1:1600 (Figure 1G).

### 3.2. Individual Recombinant Proteins Are Immunogenic but Induce Limited Functional Antibody Responses

**Figure 2:**
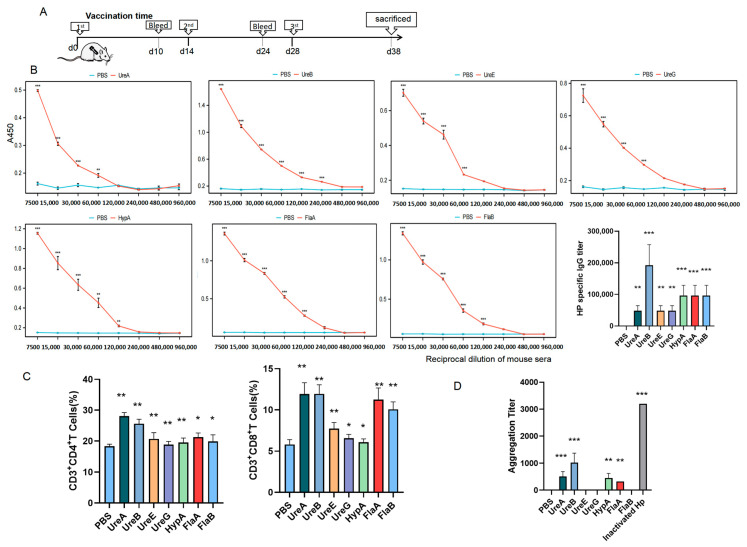
**A single bacterial protein cannot induce highly effective protective antibodies.** (**A**) Timeline diagram of immunization procedure in mice. (**B**) Titer detection of IgG antibody against each bacterial protein of *H. pylori* in immunized mouse sera by ELISA. (**C**) Ratio of CD4^+^ and CD8^+^ T cells in splenocytes detected by flow cytometry. (**D**) Agglutination titers of different immune sera to *H. pylori* (vs. PBS, * *p* < 0.05, ** *p* < 0.01, and *** *p* < 0.005; *n* = 5 in each group).

**Figure 3:**
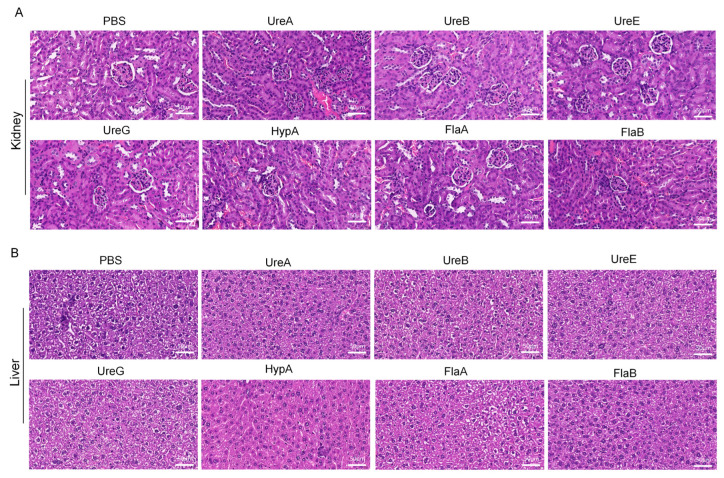
**H&E staining of kidneys and livers from mice immunized with individual recombinant proteins**. (**A**) HE staining of kidney in different subgroups. (**B**) HE staining of liver in different subgroups. Scale bar: 50 μm. (Data were obtained from mice immunized with recombinant target proteins as described in Materials and Methods.) PBS: Phosphate-buffered saline; UreA: Urease subunit A gene; UreB: Urease subunit B gene; UreE: Urease subunit E gene; UreG: Urease subunit G gene; HypA: *Helicobacter pylori* adhesin A gene; FlaA: *Helicobacter pylori* flagellin A gene; FlaB: *Helicobacter pylori* flagellin B gene.

To evaluate the immunogenicity and safety of key immunogens, recombinant UreA, UreB, UreE, UreG, HypA, FlaA, and FlaB were successfully expressed and purified (Figure S1B). Then, mice were immunized with each protein (Figure 2A) according to the dosing strategy described in Materials and Methods, and specific responses of total IgG antibody, as well as IgA, were detected in serum specimens of the immunized mice. As shown in Figure 2B, all seven proteins were immunogenic in mice and induced special IgG antibodies. Particularly, sera from mice immunized with UreB or HypA or FlaA or FlaB acquired a stronger binding to total bacterial proteins than those immunized with the other proteins, whereas the control samples from PBS-immunized mice revealed negligible binding activity (Figure 3A), confirming the specificity of such IgG antibody response elicited by these recombinant proteins. Meanwhile, the results of flow cytometry indicated that UreA and UreB, as well as FlaA and FlaB, stimulated significant T-cell activation, which provided support for the production of specific antibodies (Figure 2C). Subsequently, we conducted an agglutination reaction using these sera with *H. pylori*, and the results showed that UreA, UreB, HypA, and FlaA demonstrated significant abilities to bind to the bacteria, with UreB being the most potent (Figure 2D). No hepatorenal toxicity was observed in mice immunized with any single protein (Figure 3A,B).

### 3.3. A Multi-Antigen Cocktail Synergistically Elicits Highly Specific of Antibodies Against H. pylori

**Figure 4:**
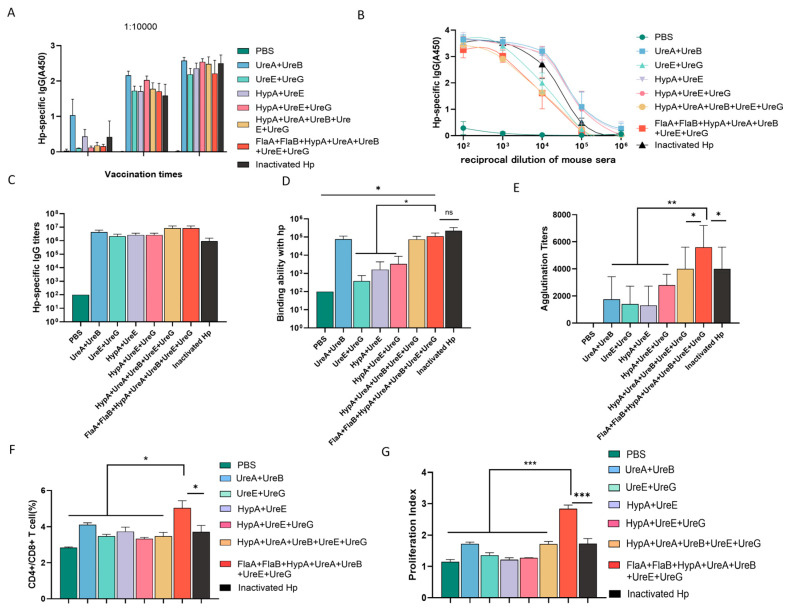
**Flagellin synergizes with urease subunits to elicit highly protective IgG antibodies against *H. pylori.*** (**A**) Serum IgG titer dynamics in mice immunized with different recombinant protein combinations. (**B**,**C**) Hp-specific IgG levels in the sera of different group. (**D**) Specific binding capacity of immune sera to *H. pylori* whole-cell lysate. (**E**) Agglutination assay analysis of the protective antibody titers against *H. pylori* in the immune sera from each group. (**F**) Ratio of CD4^+^ T/CD8^+^ T cells in splenocytes detected by flow cytometry. (**G**) Proliferation of spleen cells was detected by flow cytometry (* *p* < 0.05, ** *p* < 0.01, and *** *p* < 0.005; *n* = 5 in each group).

Although individual recombinant protein has been shown to generate efficient IgG specific to *H. pylori* and elicit adaptive immunity in mice, some ureases from *H. pylori* can form complexes when combined. Notable examples of these complexes include the UreA-UreB complex [21], the UreE-UreG complex [22,23,24], and the HypA-UreE complex [25]. These ureases may form complexes spontaneously or through competitive binding interactions. Building on this premise, we evaluated various protein combinations. As illustrated in Figure 4A, administration of two booster immunization doses led to a progressive increase in serum IgG titers across all groups, with minimal differences observed in the overall IgG levels induced among the different vaccination regimens (Figure 4B,C). However, substantial variations emerged in the capacity of immune sera from each group to specifically bind to the whole-cell lysate of *H. pylori*. Sera from groups immunized with UreA + UreB, HypA + UreA + UreB + UreE + UreG, and the comprehensive FlaA + FlaB + urease subunit cocktail showed the strongest binding to *H. pylori* whole-cell lysate, and no statistically significant differences were detected among them. In contrast, sera from the HypA + UreE, UreE + UreG, and HypA + UreE + UreG groups exhibited negligible high-affinity binding to the whole-cell lysate of *H. pylori* (Figure 4D). Crucially, the FlaA + FlaB + HypA + UreA + UreB + UreE + UreG combination induced the highest agglutination titer (approximately 7 × 10^3^), numerically exceeding that of the inactivated whole *H. pylori* group (Figure 4E). Notably, this same combination also promoted robust activation and proliferation of CD4^+^ T lymphocytes (Figure S2A; Figure 4F,G). Together, these findings indicate that the co-administration of urease-associated proteins with flagellin subunits acts synergistically to effectively activate both humoral and cellular immune pathways, eliciting high-titer, specific IgG antibodies with binding capacity against *H. pylori* that is comparable or superior to that induced by inactivated whole-cell vaccine.

### 3.4. Combination Immunization with Candidate Proteins Shows No Hepatorenal or Splenic Toxicity

**Figure 5:**
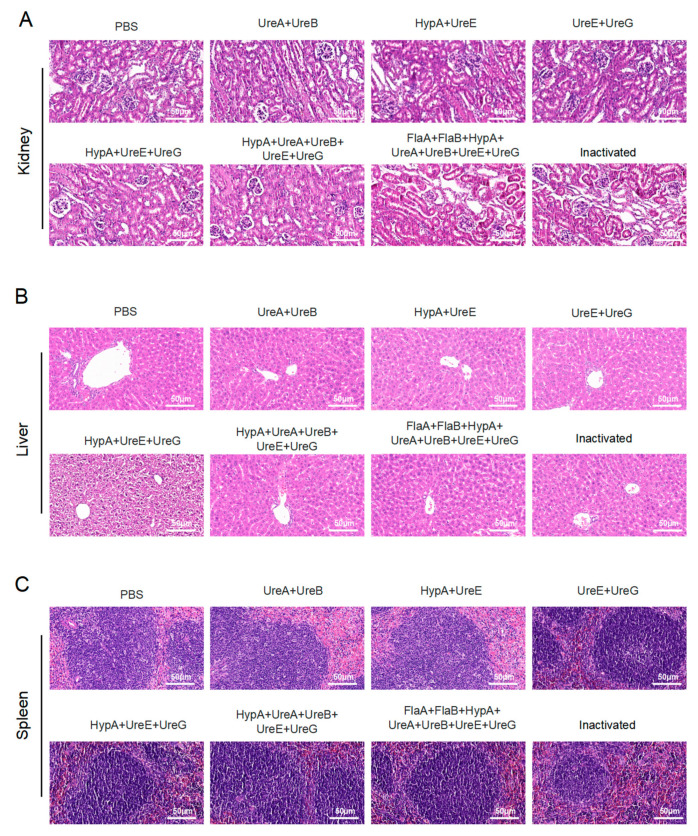
**Safety evaluation of candidate vaccines in immunized mouse models.** (**A**) H&E staining of kidney from mice immunized with multiple recombinant proteins. (**B**) H&E staining of liver from mice immunized with multiple recombinant proteins. (**C**) H&E staining of spleen from mice immunized with multiple recombinant proteins. Scale bar: 50 μm. (Data were obtained from mice immunized with recombinant target proteins as described in Materials and Methods.) PBS: Phosphate-buffered saline; UreA: Urease subunit A gene; UreB: Urease subunit B gene; UreE: Urease subunit E gene; UreG: Urease subunit G gene; HypA: *Helicobacter pylori* adhesin A gene; FlaA: *Helicobacter pylori* flagellin A gene; FlaB: *Helicobacter pylori* flagellin B gene.

Histopathological evaluation of liver, kidney, and spleen tissues harvested from immunized mice revealed no significant morphological alterations or signs of toxicity across all candidate polyprotein vaccine groups. H&E staining demonstrated tissue architectures comparable to those of PBS-administered control animals, with no evidence of inflammation, necrosis, or other pathological changes. These findings indicate that immunization with the various protein combinations did not induce detectable hepatic, renal, or splenic toxicity, supporting the favorable safety profile of the tested Hp subunit vaccine formulations (Figure 5A–C).

## 4. Discussion

### 4.1. The Challenge of Antimicrobial Resistance and the Need for Novel Strategies

*H. pylori* is a globally prevalent pathogen, colonizing nearly half of the world’s population. Its persistent infection is a major etiological factor in a spectrum of upper gastrointestinal disorders, including chronic dyspepsia, gastritis, peptic ulcer disease, and, notably, gastric adenocarcinoma [4]. Consequently, the successful eradication of *H. pylori* is a well-established clinical strategy for halting disease progression and preventing severe complications. However, the escalating crisis of antimicrobial resistance (AMR), fueled largely by the irrational and widespread use of antibiotics, has severely compromised the efficacy of standard eradication regimens, posing a formidable challenge to global *H. pylori* management. The World Health Organization has rightfully declared AMR a top-tier global public health threat. The staggering burden—approximately 495 million infections and 1.27 million deaths directly attributed to bacterial AMR in 2019 alone—underscores the urgency of the situation [26]. Projections that AMR-related mortality could eclipse cancer deaths by 2050, reaching 10 million annually, further amplify the call for immediate action. Within this critical context, the development of effective, non-antibiotic therapeutic alternatives for *H. pylori* infection is not merely an academic pursuit but an urgent public health imperative.

The landscape of non-antibiotic approaches in China, while promising, remains in nascent stages of development and application. An analysis of patent filings reveals fewer than twenty applications specifically for non-antibiotic *H. pylori* treatments, with a predominant focus on prophylactic vaccines. A landmark achievement was the urease-based oral recombinant vaccine developed by Zeng et al., which progressed through phase III clinical trials. Although it demonstrated an initial protective efficacy of 70% in a pediatric cohort, this effect waned to 56% over one year [27]. This highlights a common hurdle in vaccine development: achieving durable, high-level protection. Other vaccine candidates under investigation, while showing immunogenicity, have yet to meet desired efficacy benchmarks. Parallel exploration in traditional medicine, including compounds like synthetic linolenic acid which showed promise comparable to triple therapy, offers intriguing avenues [28]. However, the transition from traditional use to evidence-based therapy requires rigorous, large-scale clinical validation of safety, efficacy, and standardization. Similarly, adjuvant use of probiotics has shown benefit, particularly in improving tolerability and outcomes in specific populations like elderly ulcer patients, yet it fundamentally remains an adjunct to, not a replacement for, antibiotic therapy, thus not solving the core resistance problem [29,30]. Collectively, these efforts underscore a significant unmet need for a novel, effective, and scalable non-antibiotic intervention that can directly counteract the pathogen.

### 4.2. Bovine Immune Milk: A Stable Platform for Functional Antibody Delivery

Our first key finding establishes the feasibility of bovine immune milk as a source of stable, functional antibodies against *H. pylori* [14]. The IgG antibodies produced not only recognized major virulence factors but, importantly, retained agglutination activity under acidic conditions mimicking the gastric environment (Figure 1C) This acid stability is a critical prerequisite for any orally delivered antibody therapeutic targeting a gastric pathogen. The long-term stability of these antibodies, maintained over three years of storage, further underscores the practical potential of this platform for developing a shelf-stable, orally administrable product. Such a product could be particularly valuable for vulnerable populations, such as infants or immunocompromised individuals, as a means of passive immunological protection.

### 4.3. Rational Antigen Selection and Synergistic Immunogen Design

The proteomic profiling of immune milk antibodies provided a data-driven blueprint for antigen selection, highlighting urease subunits and flagellins as dominant targets. Translation of this finding into a murine active immunization model yielded a second, significant insight: while individual proteins were immunogenic, their functional antibody output, as measured by agglutination titer, was limited.

The synergistic effect observed with the multi-protein cocktail—particularly the combination incorporating both flagellins and the suite of urease-associated proteins—represents the core advance of this study. This formulation induced agglutination titers that surpassed those elicited by inactivated whole bacteria (Figure 4E). We posit that this synergy may result from several mechanisms: (i) The subunit cocktail provides a more concentrated and focused presentation of key protective antigens compared to the complex and diluted mixture of antigens present in whole bacterial lysates, potentially leading to higher overall immunogenicity against selected virulence factors. (ii) This approach delivers a broader, rationally designed array of epitopes, engaging a more diverse B-cell repertoire and promoting a polyclonal antibody response with broader specificity. (iii) Such multi-antigen exposure may facilitate improved antibody affinity maturation through enhanced T-cell help directed against a wider spectrum of antigens. (iv) The co-delivery of proteins known to interact in vivo (e.g., urease subunits) may better mimic native functional complexes, potentially inducing antibodies with higher functional avidity. The concurrent robust CD4^+^ T-cell activation supports the engagement of effective helper T-cell responses (Figure 4F,G).

### 4.4. Limitations and Future Directions

The current study is fundamentally an immunogenicity and antigen optimization investigation. While agglutination is a valuable in vitro correlate of antibody function, it does not directly equate to in vivo protection or bacterial clearance. The promising immunogenicity and excellent safety profile of the multi-antigen cocktail justify and necessitate further investigation. Further work will focus on direct functional validation, including the following: 1. In vivo Challenge Models: Determining the efficacy of immune milk or sera from multi-antigen-immunized animals in reducing bacterial colonization, gastric inflammation, and pathology in established animal models of *H. pylori* infection. 2. Formulation and Delivery Optimization: Exploring adjuvants more suitable for human use, alternative delivery routes (e.g., mucosal), and the development of scalable production processes for the antigen cocktail.

### 4.5. Translational Potential and Path Forward: Immune Milk Maybe Act as a Therapeutic or Prophylactic Product

The stable, orally deliverable nature of immune milk positions it as a potential dietary supplement or a “nutraceutical” intervention. It could serve as an adjunct to standard therapy to improve eradication rates or as a prophylactic measure in high-risk settings. Engagement with food technology and dairy industry partners would be crucial for scaling production, standardizing antibody titers, and navigating the regulatory landscape, which may differ from that of pharmaceuticals.

## 5. Conclusions

In summary, this study demonstrates that bovine immune milk can serve as a robust source of acid-stable, multi-target antibodies against *H. pylori*. Furthermore, by employing a reverse vaccinology approach informed by the immune milk antibody repertoire, we have identified a specific multi-antigen combination that synergistically enhances functional humoral immune responses in mice. These findings provide a rational and promising foundation for the future development of both antibody-based and vaccine-based non-antibiotic strategies against *H. pylori*. Subsequent research must bridge the gap between these compelling immunogenicity results and demonstrated efficacy in functional and protective models.

## Data Availability

The data that supports the findings of this study are available within the article. The original data can be obtained by sending an email to the corresponding author.

## Supplementary Materials

The following supporting information can be downloaded at: https://www.mdpi.com/article/10.3390/vaccines14020110/s1, Figure S1: Purification of each bacteriophage protein; Figure S2: The ratio of CD4^+^ to CD8^+^ lymphocytes in splenic lymphocytes from mice immunized with multiple antigens.
