# Supplementary figures and images for: From Bovine Immune Milk Profiling to Multi-Antigen Vaccine Design: Enhanced Humoral Responses Against H. pylori with a Flagellin and Urease Subunit Cocktail
Source: Vaccines (Basel). 2026 Jan 23;14(2):110. doi: 10.3390/vaccines14020110 (PMC12945149; doi:10.3390/vaccines14020110)

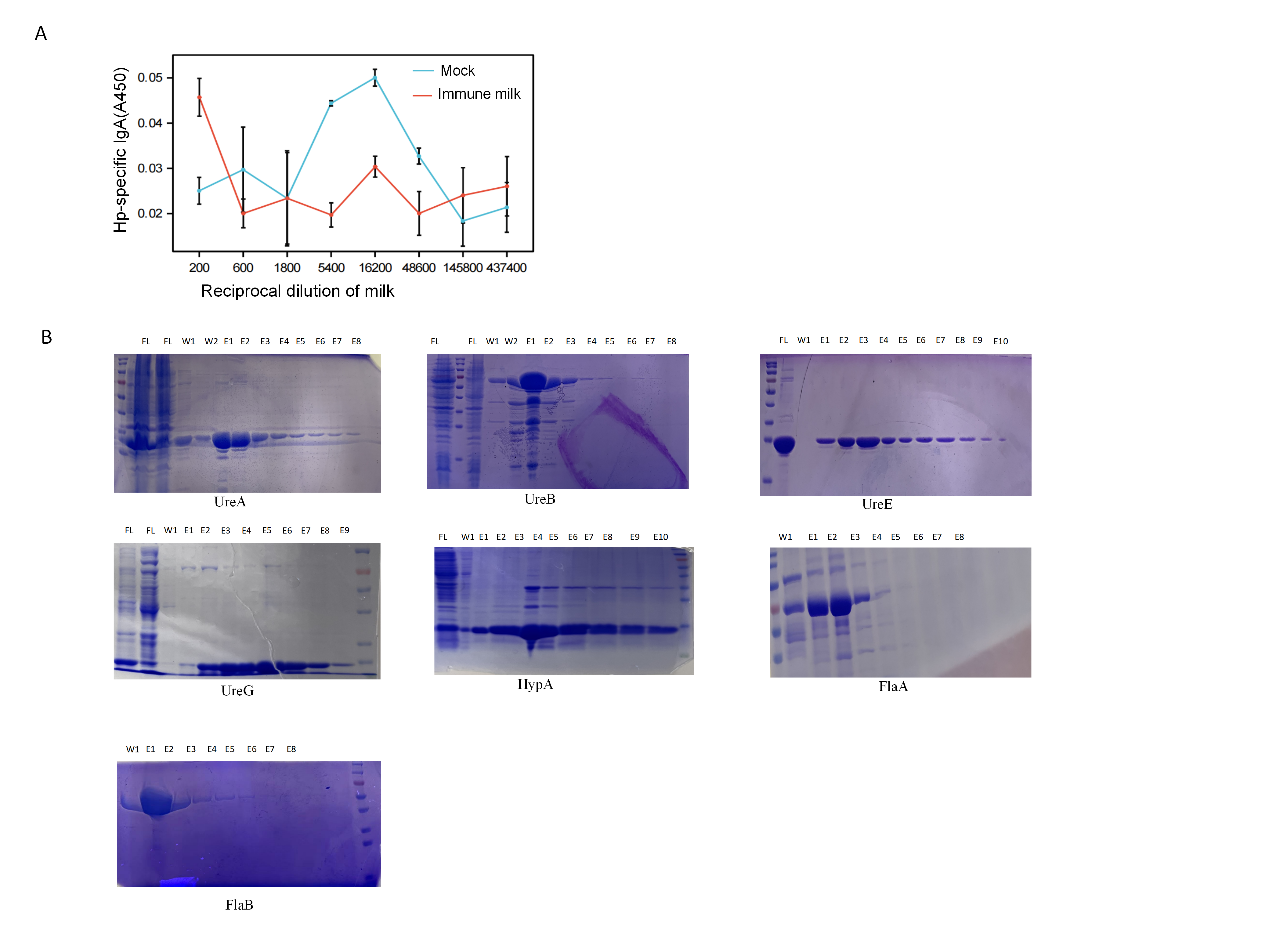

Supplement: Supplementary file 1 [file vaccines-14-00110-s001.zip › Figure S1.tif]

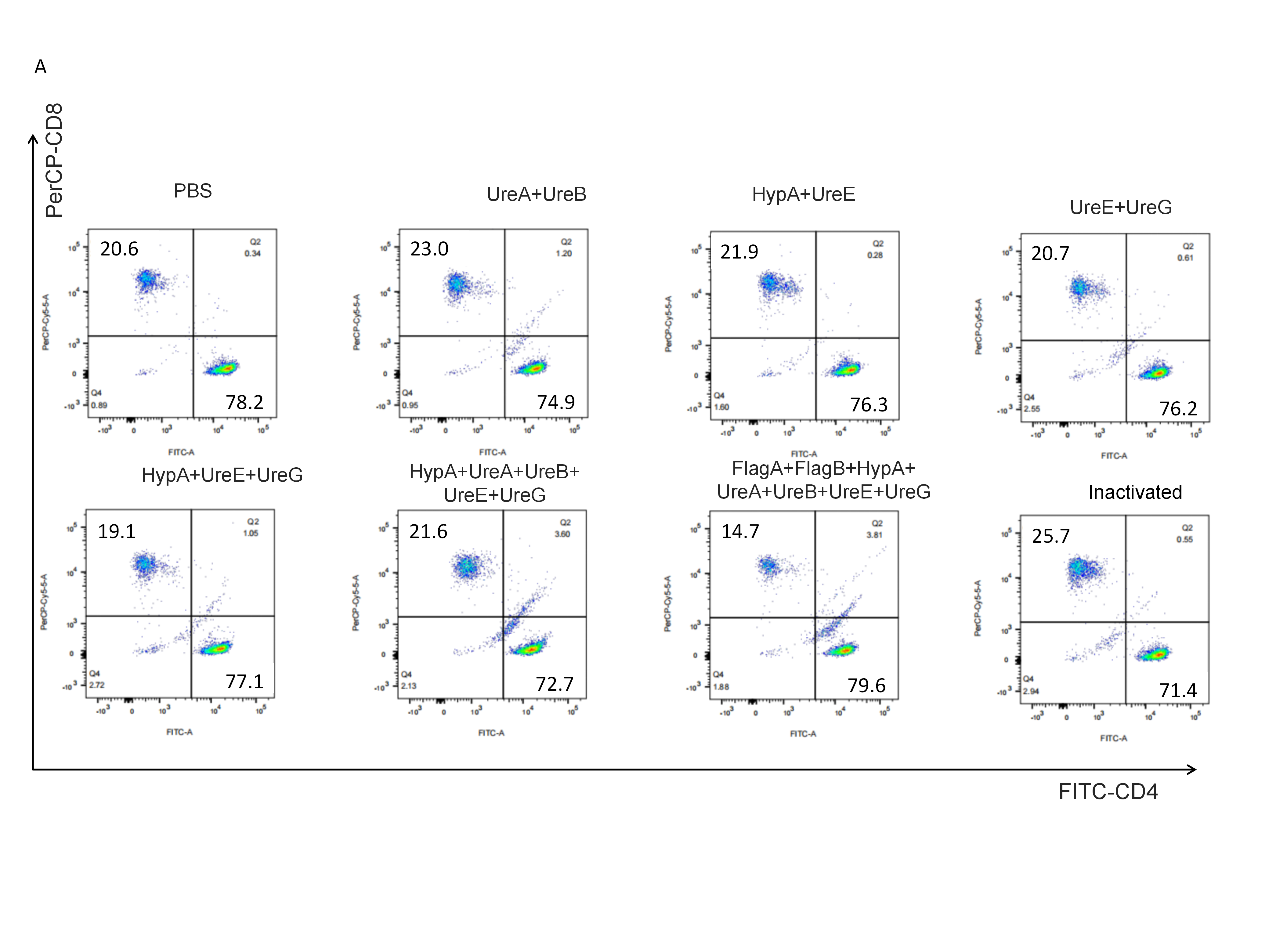

Supplement: Supplementary file 1 [file vaccines-14-00110-s001.zip › Figure S2.tif]
